# Using CMR to improve the diagnostic accuracy of the ECG for the detection of left ventricular hypertrophy; production of a simple adjustment for body mass index

**DOI:** 10.1186/1532-429X-18-S1-Q35

**Published:** 2016-01-27

**Authors:** Oliver Rider, Sacha C Bull, Richard M Nethononda, Ntobeko A Ntusi, Vanessa M Ferreira, Cameron Holloway, David A Holdsworth, Masliza Mahmod, Jennifer J Rayner, Rajarshi Banerjee, Saul G Myerson, Stefan Neubauer, Hugh Watkins

**Affiliations:** grid.4991.50000000419368948Cardiovascular Medicine, University of Oxford, Oxford, United Kingdom

## Background

The electrocardiogram (ECG) is the most widely used screening tool to detect left ventricular hypertrophy (LVH). However, current diagnostic criteria are highly insensitive, particularly in modern society with increasing prevalence of obesity. We aimed to use cardiac magnetic resonance (CMR) imaging to assess anatomical LV axis (frontal and lateral axis) and LV mass across a wide range of different body sizes, to generate a simple and clinically applicable adjustment factor that could be applied to increase the sensitivity of the most commonly used diagnostic criteria.

## Methods

A single centre, retrospective study of 1295 participants. An initial cohort (n = 821, ♂n = 450, ♀n = 371, aged 19-87 years) with a wide range of body mass index (BMI, 17.1-53.3 kg/m^2^) underwent CMR evaluation (1.5T) to determine anatomical LV axis and LV mass, as well as a standard 12-lead surface ECG. The anatomical LV axis was defined as the plane between the centre of the mitral valve and the LV apex using multi-planar reconstruction of thoracic Half fourier Acquisition Single shot Turbo spin Echo (HASTE) images within cmr42 ©. LVH was defined as >2SD higher than the mean of the normal Oxfordshire population from which the study sample was taken (>165 g in males, >150 g in females). The effect of BMI and LV axis on Sokolow-Lyon index (S in V_1_ + R in V_5_/V_6_ ≥ 35 mm) and Cornell criteria (S in V3 + R in aVL >28 mm (♂) or > 20 mm (♀)) were then calculated and an adjustment factor generated. A second validation cohort (n = 474, BMI 15.9-63.2 kg/m^2^) underwent CMR evaluation (1.5T) to determine LV mass, and standard 12-lead surface ECG. Sensitivity and specificity for the ECG detection of CMR derived LVH was then determined for the study groups with and without adjustment.

## Results

**Figure 1:**
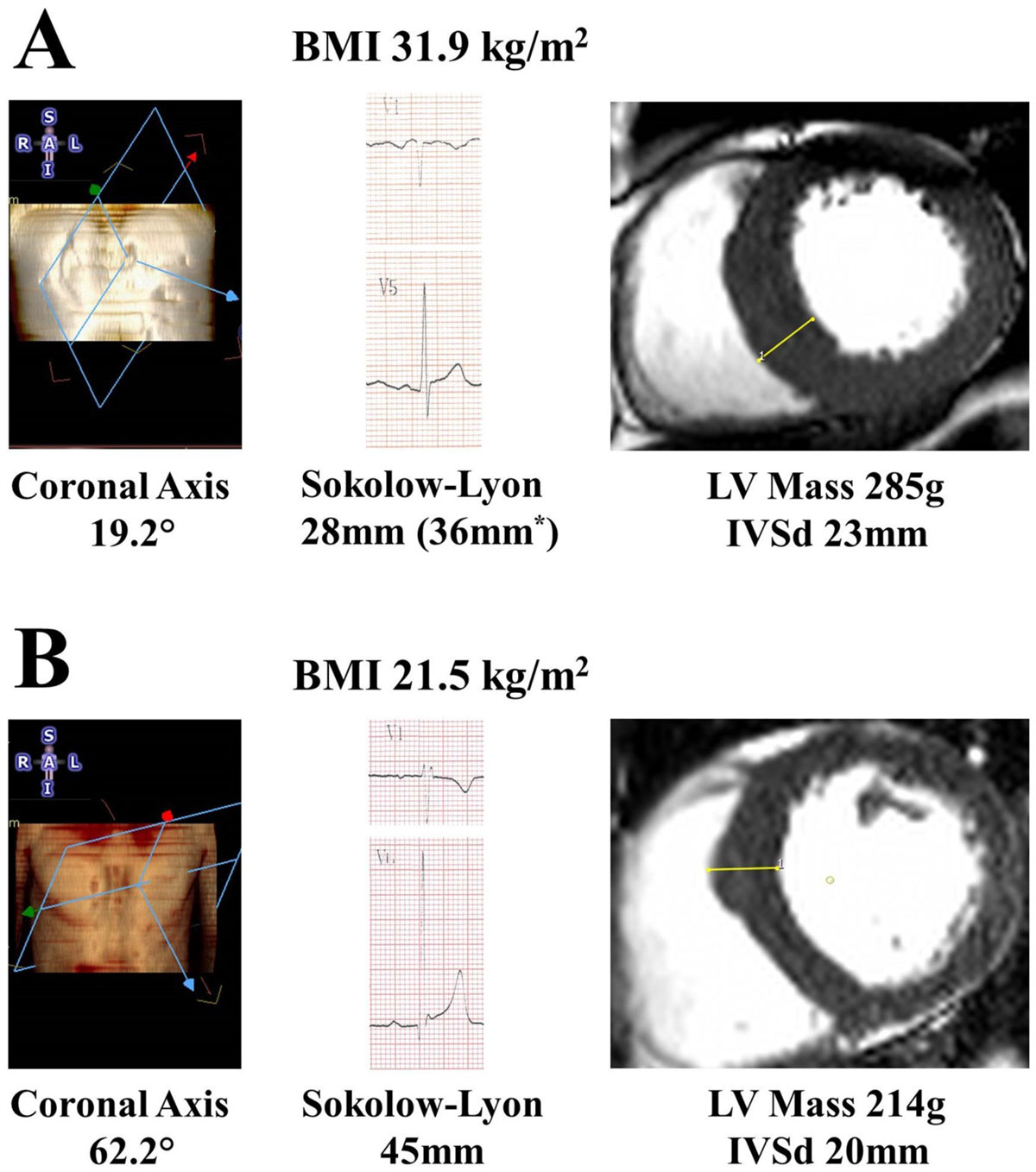
**Improving the diagnostic accuracy of the surface ECG**.

When matched for LV mass, the combination of leftward anatomical axis deviation and increased BMI resulted in a reduction of the Sokolow-Lyon index, by 4 mm in overweight and 8 mm in obesity (Figure 1). After adjusting for this in the initial cohort, the sensitivity of the Sokolow-Lyon index dramatically increased (overweight 12.8% to 31.9%, obese 2.9% to 27.2%) approaching that seen in normal weight (37.8%). Similar results were achieved in the validation cohort (specificity increase overweight 21.1% to 42.1%; obese 6.3% to 21.9%) again approaching normal weight (33.3%). Importantly, specificity remained excellent (>96.0%).The Cornell criteria although less affected by BMI, had poor sensitivity (14.8%), and were not amenable to adjustment.

## Conclusions

By using CMR to assess LV mass and anatomical axis deviation we have generated a simple BMI based clinically relevant adjustment factor for the surface ECG. Adjusting the Sokolow-Lyon index for BMI (overweight +4 mm, obesity +8 mm) dramatically improves the diagnostic accuracy for detecting LVH. As the ECG, worldwide, remains the most widely used screening tool for LVH, implementing these findings should translate into significant clinical benefit.

